# Brain-Sparing Methods for IMRT of Head and Neck Cancer

**DOI:** 10.1371/journal.pone.0120141

**Published:** 2015-03-17

**Authors:** Alex Dunlop, Liam Welsh, Dualta McQuaid, Jamie Dean, Sarah Gulliford, Vibeke Hansen, Shreerang Bhide, Chris Nutting, Kevin Harrington, Kate Newbold

**Affiliations:** 1 Joint Department of Physics, Institute of Cancer Research and The Royal Marsden NHS Foundation Trust, London, United Kingdom; 2 The Royal Marsden Hospital, London, United Kingdom; 3 The Institute of Cancer Research, London, United Kingdom; University of California Davis, UNITED STATES

## Abstract

**Purpose:**

Radical radiotherapy for head and neck cancer (HNC) may deliver significant doses to brain structures. There is evidence that this may cause a decline in neurocognitive function (NCF). Radiation dose to the medial temporal lobes, and particularly to the hippocampi, seems to be critical in determining NCF outcomes. We evaluated the feasibility of two alternative intensity-modulated radiotherapy (IMRT) techniques to generate hippocampus- and brain-sparing HNC treatment plans to preserve NCF.

**Methods and Materials:**

A planning study was undertaken for ten patients with HNC whose planning target volume (PTV) included the nasopharynx. Patients had been previously treated using standard (chemo)-IMRT techniques. Bilateral hippocampi were delineated according to the RTOG atlas, on T1w MRI co-registered to the RT planning CT. Hippocampus-sparing plans (HSRT), and whole-brain/hippocampus-sparing fixed-field non-coplanar IMRT (BSRT) plans, were generated. DVHs and dose difference maps were used to compare plans. NTCP calculations for NCF impairment, based on hippocampal dosimetry, were performed for all plans.

**Results:**

Significant reductions in hippocampal doses relative to standard plans were achieved in eight of ten cases for both HSRT and BSRT. EQD2 D40% to bilateral hippocampi was significantly reduced from a mean of 23.5 Gy (range 14.5–35.0) in the standard plans to a mean of 8.6 Gy (4.2–24.7) for HSRT (p = 0.001) and a mean of 9.0 Gy (4.3–17.3) for BSRT (p < 0.001). Both HSRT and BSRT resulted in a significant reduction in doses to the whole brain, brain stem, and cerebellum.

**Conclusion:**

We demonstrate that IMRT plans for HNC involving the nasopharynx can be successfully optimised to significantly reduce dose to the bilateral hippocampi and whole brain. The magnitude of the achievable dose reductions results in significant reductions in the probability of radiation-induced NCF decline. These results could readily be translated into a future clinical trial.

## Introduction

Around two-thirds of patients with head and neck cancer (HNC) present with locally advanced disease, and the majority receive radical chemo-radiotherapy (RT) [1–3]. Intensity-modulated radiation therapy (IMRT) developments over the last decade have improved dose-sparing of organs-at-risk (OARs) whilst maintaining, or even escalating, planning target volume (PTV) dose [4, 5]. IMRT for HNC is, however, associated with a low dose bath to normal tissues, notably the brain [6, 7], and this has been associated with increased acute neurotoxicity in the PARSPORT randomised trial of parotid-sparing IMRT for HNC [4]. Analysis of dosimetric data from the PARSPORT trial shows that the excess fatigue in the IMRT arm is associated with increased dose to the posterior fossa, and specifically to the cerebellum [6].

Whether this low dose radiation bath to the brain causes long-term neurotoxicity is unclear. Radiation-induced brain injury (RIBI) due to conventional radiotherapy for HNC, especially nasopharyngeal carcinoma (NPC), has long been associated with late neurotoxicity, including temporal lobe necrosis and decreased neurocognitive function (NCF) [8–10]. Impaired NCF following brain irradiation is positively associated with radiation dose to the temporal lobes and, more specifically, with dose to the hippocampi [11–14]. Prospective NCF outcome data from an RTOG study of fractionated brain radiotherapy for benign and low-grade brain tumours found that a biologically equivalent dose in 2 Gy fractions (EQD2) to 40% (D40%) of the bilateral hippocampi greater than 7.3 Gy (assuming an α/β ratio of 2 Gy) is associated with the development of NCF impairment [13]. A normal tissue complication probability (NTCP) model for NCF impairment following hippocampal irradiation was developed from these data [13]. Allowing for differences in fractionation, typical IMRT plans for HNC may result in hippocampal doses of sufficient magnitude to exceed this NTCP-modelled threshold, leading to a high probability of NCF impairment [6, 7, 11, 12]. The limited available clinical data do demonstrate small, but statistically significant, declines in NCF following modern (chemo)-IMRT for NPC [11], and other HNC sites [12]. However, the clinical significance of these observed declines in NCF remains unclear and further prospective studies are needed to address this issue [24].

There is increasing evidence for differential radiosensitivity across different anatomical brain regions [14–16]. Within the medial temporal lobes, neural stem cells in the subgranular zone of the hippocampal dentate gyrus have been shown to be exquisitely radiosensitive, and radiation dose to this brain region predicts for subsequent NCF impairment, and specifically for reduced short-term memory function [15, 17, 18]. There is, therefore, a strong rationale for minimising radiation dose to the hippocampus during radiotherapy for HNC to preserve NCF [17]. This hippocampus-sparing hypothesis has recently received additional support from the results of the RTOG 0933 phase II study of hippocampus-sparing WBRT for patients with brain metastases [19].

The low dose radiation bath to the brain also disrupts the blood-brain-barrier [20, 21]. Therefore, it has potential to increase brain exposure to concomitantly delivered chemotherapy agents, such as cisplatin, which have significant neurotoxicity [22, 23]. Thus, in order to minimise radiotherapy induced blood-brain barrier disruption, and thereby neurotoxicity from concomitant chemotherapy, it may be important to minimise the whole brain radiation dose, over and above the additional specific need to spare the hippocampi.

Minimising RT dose to the brain, and particularly to the hippocampi, is an obvious strategy to reducing the risk of treatment-induced NCF impairment due to (chemo)-IMRT for HNC [24]. Here, we present two distinct photon-based IMRT planning approaches to reducing brain dose for HNC patients whilst maintaining clinically acceptable plans. The first approach, termed hippocampus-sparing RT (HSRT) aims specifically to reduce the hippocampal RT dose. The second approach, termed brain-sparing RT (BSRT), aims to reduce the whole brain RT dose in addition to sparing the hippocampi. By sparing the whole brain, this latter approach might achieve reductions in acute and late neurotoxicities (eg NCF).

## Materials and Methods

### Patient characteristics

This is a non-interventional retrospective planning study in adult patients with HNC. Written informed consent was obtained from all patients. This research was approved by the institutional review board (Royal Marsden Hospital Committee for Clinical Research CCR3767) and research ethics committee (NHS REC number 10/H0801/32). Ten patients with HNC that were treated with radical radiotherapy to a PTV that included the nasopharynx were chosen for study. The indication for nasopharyngeal irradiation was either as part of treatment for a primary tumour within the nasopharynx (NPT; 8 patients), or as part of total mucosal irradiation for a head and neck squamous cell carcinoma of unknown primary origin (SCCUP; 2 patients). This HNC patient cohort was selected on the basis that patients undergoing nasopharyngeal irradiation often receive significant radiation doses to the brain. Characteristics of the patients are given in Table 1. Patients were treated with (chemo)-IMRT, as previously described [25], to doses of 65 Gy and 54 Gy in 30 fractions over 6 weeks, prescribed to the mean of the primary target including involved nodes (PTV1), and the elective target including non-involved neck nodes (PTV2), respectively.

**Table 1:** Patient characteristics.

Patient	Sex	Age	Primary Site	Histology	T	N	Absolute mean brain dose (Gy)	Absolute mean bilateral hippocampal dose (Gy)	Mean bilateral hippocampal EQD2 (Gy)*	Bilateral hippocampal EQD2 D40% (Gy)*
1	F	58	Nasopharynx	UCNT	4	3	16.2	38.8	31.9	35.0
2	F	27	Nasopharynx	UCNT	4	1	12.4	30.2	22.7	24.8
3	M	43	Nasopharynx	SCC	1	2	12.2	26.7	19.2	19.7
4	M	55	Nasopharynx	UCNT	1	1	12.4	26.6	19.2	21.0
5	F	62	Nasopharynx	SCC	4	0	16.9	38.5	31.6	33.9
6	M	26	Nasopharynx	UCNT	2	2	10.0	20.1	13.4	14.5
7	F	27	Nasopharynx	UCNT	1	1	9.3	20.0	13.3	16.4
8	M	20	Nasopharynx	UCNT	2	2	11.7	28.4	20.9	22.8
9	F	51	Unknown	SCC	0	2a	2.4	2.4	1.2	1.3
10	M	63	Unknown	SCC	0	2a	8.0	8.0	4.5	4.9

SCC, squamous cell carcinoma; UCNT, undifferentiated carcinoma of nasopharyngeal type.

*for α/β = 2 Gy

### RT structure delineation

**Fig 1:**
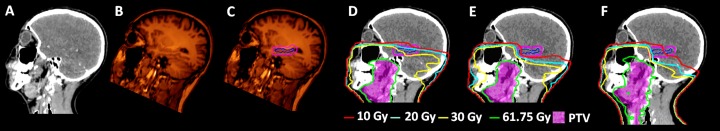
Hippocampal delineation and treatment planning. Sagittal views of (A) the RT planning CT; (B) the registered T1w image; (C) the T1w image with the left hippocampus and left hippocampal PRV shown; (D) standard clinical, (E) HSRT and (F) BSRT HNC treatment plans. In C, D, E, and F the hippocampus and hippocampal PRV are shown as blue and pink contours respectively.

Patients underwent computed tomography (CT) for IMRT planning, with 2 mm slice thickness, of the entire head and neck region using thermoplastic mask immobilisation. T1-weighted (T1w) magnetic resonance imaging (MRI) with 2 mm slice thickness was acquired over the same anatomical region. CT and T1w MRI images were manually rigidly co-registered using the Philips Pinnacle^3^ version 9.0 (Philips, Fitchburg, WI) radiotherapy treatment planning system (TPS). The hippocampi were delineated on the T1w MRI according to the RTOG hippocampal atlas [13, 28]. Hippocampal planning organ-at-risk volumes (PRVs) were generated by isotropically expanding the hippocampi by 5 mm in accordance with the RTOG 0933 protocol (Fig. 1) [26]. Clinical target volumes (CTVs), and organ-at-risk volumes (OARs), were delineated on the planning CT according to standard institutional protocols. For NPT patients, the entire sphenoid sinus was included in CTV1 for T4 disease with skull base invasion; in the absence of T4 skull base involvement, the superior half of the sphenoid sinus was included in CTV2, and the inferior half in CTV1. The sphenoid sinus was not included within the CTV for patients with SCCUP. CTVs were isotropically expanded by 3 mm to generate PTVs. Edited PTVs were generated for plan evaluation and treatment prescription, defined as the PTV excluding any tissue within 5 mm of the external body contour. In addition, OARs for the cerebellum, brainstem, temporal lobes, and cochleae were delineated according to institutional protocols by an experienced radiation oncologist (LW). According to recommendations from the International Commission on Radiological Units and Measurements (ICRU), the remaining volume at risk (RVR) was defined as the remaining non-contoured normal tissue [27].

### Planning objectives and techniques

All radiotherapy plans were generated and optimised using Philips Pinnacle^3^ v9.0 TPS. Standard clinical plans used for treatment were generated according to institutional protocols using either fixed-field step-and-shoot IMRT (n = 2), or a single VMAT beam (n = 8). Clinical VMAT plans consisted of 180 control points with 2° control point spacing, optimised using Pinnacle’s SmartArc algorithm. Clinical fixed-field IMRT plans consisted of between 5 and 7 co-planar beams with a maximum of 60 control points in total. Two additional brain-sparing plans were subsequently generated for each of the 10 patients: 1) a hippocampus-sparing (HSRT) plan, and 2) a whole brain-sparing fixed-field non-coplanar IMRT (BSRT) plan.

Pinnacle’s Direct Machine Parameter Optimisation (DMPO) algorithm and dose engine was used to optimise the shapes and weights of the individual apertures of the fixed-field IMRT plans. In order to avoid the optimiser boosting target tissue within the build-up region of the patient, virtual bolus (density = 1.0 g/cm^3^) was generated exterior to the body contour such that PTV1 and PTV2 were always at least 1 cm from either the external body contour or the virtual bolus surface. Plans were optimised using bespoke objective functions for each patient to generate optimal plans and to satisfy clinical dose objectives (S1 Table). Once a satisfactory plan was generated, virtual bolus was removed and the dose prescribed to the edited PTV volumes.

To generate HSRT plans, additional optimisation objectives, for both maximum and mean doses, were used for the bilateral hippocampi, and the left and right hippocampal PRVs. The maximum dose objectives were determined from the distance between the OAR and the PTV, whilst a mean dose of <12 Gy was initially set for both OARs. The beam arrangement used for HSRT was as for the clinical plans. HSRT plans were optimised to achieve the lowest hippocampal dose while maintaining clinically acceptable PTV coverage and OAR sparing. BSRT treatment plans were generated using a fixed-field IMRT technique consisting of between 6–8 non-coplanar beams, including anterior superior oblique, and posterior inferior oblique, beams designed to avoid large regions of the brain, including the bilateral hippocampi (S1 Fig.). To ensure that the HSRT and BSRT plans were deliverable with sufficient accuracy, a subset of these plans (2 of each, chosen at random) were verified using the Delta4 (ScandiDos, Uppsala, Sweden) phantom, with a global gamma criterion of 3%/3 mm. Plans were deemed acceptable if at least 95% of points exhibited a gamma index of <1.

### Treatment plan evaluation and data analysis

Along with the clinical dose statistic objectives (S1 Table), additional parameters were used to evaluate the standard clinical, HSRT, and BSRT radiotherapy plans including the dose to the bilateral hippocampi, whole brain, cerebellum, temporal lobes, cochleae, mandible, and RVR. The homogeneity index (HI) quantifies dose homogeneity in the target volume and is defined as HI = (D_2%_—D_98%_)/D_median_, where D_2%_ and D_98%_ are the maximum doses to 2% and 98% of the target volume, respectively. Smaller HI values correspond to more homogeneous plans with HI = 0 corresponding to absolute homogeneity within the target volume. The Paddick conformity index (CI) was used to quantify how well the dose distribution conformed to the size and shape of the target, with CI = ([TV(PIV)]^2^)/[TV*V(RI)], where TV(PIV), TV, and V(RI) are the volumes of the target covered by the 95% isodose, the target volume, and the total volume covered by the 95% isodose, respectively [28]. A perfectly conformal plan corresponds to CI = 1, and smaller values of CI represent less conformal dose distributions. Dose cubes were exported from Pinnacle to the RayStation TPS (RaySearch, Stockholm, Sweden) to generate dose difference maps.

Following Gondi et al. [13], we calculated the EQD2 D40% for the bilateral hippocampi (assuming α/β = 2 Gy), to evaluate the risk of treatment-induced NCF impairment for the clinical, HSRT, and BSRT plans. The effect of changes in the hippocampal EQD2 D40% on the probability of NCF impairment was evaluated using the NTCP model of NCF impairment derived from adult patients treated with fractionated stereotactic RT for benign or low-grade primary brain tumours [13]. This model relates the probability of a decline in short-term memory function, as measured by the Wechsler Memory Scale-III Word Lists delayed recall at 18 months post-RT, to the bilateral hippocampal EQD2 D40%. The effects of HSRT and BSRT on doses to the posterior fossa and cerebellum, which may relate to the incidence of acute fatigue during IMRT, were analysed by comparison of DVHs and by calculating the OAR volume receiving at least 20 Gy (V20Gy). The V20Gy dose metric reflects changes in the low dose bath received by these structures.

Statistical data analysis was performed using R (R Foundation for Statistical Computing, Vienna, Austria). Two-sided paired t-tests were used to compare mean dose metrics for standard clinical, HSRT, and BSRT plans, with a statistical significance level of alpha = 0.05.

## Results

**Fig 2:**
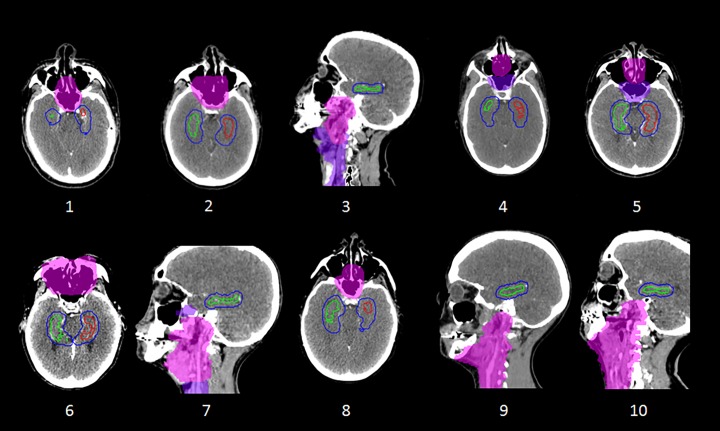
Disposition of PTVs and hippocampal PRVs for the 10 cases. Case numbering is as per Table 1. PTV1 is shown as a light pink colourwash, PTV2 as a purple colourwash, while the left hippocampus, right hippocampus and hippocampal PRVs are illustrated as red, green, and blue contours respectively. Axial or sagittal views are shown for each case according to the plane that transects both volumes.

**Fig 3:**
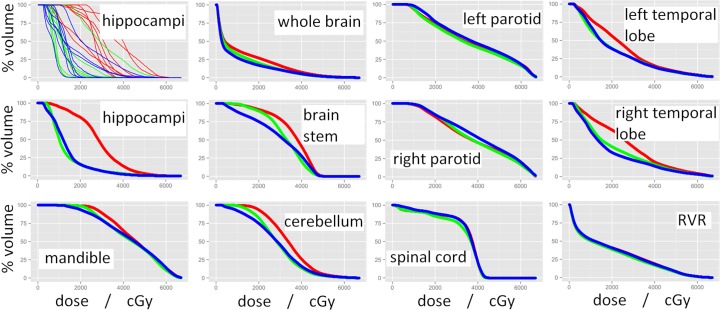
Dose-volume histograms (DVHs) for various OARs with red, green, and blue representing standard clinical, HSRT, and BSRT plans respectively. All curves shown are average DVHs for the 8 NPT cases that were re-planned using both HSRT and BSRT methods, except for the top-left panel, which shows the individual DVH curves for each patient.

The proximities of the hippocampi PRVs to the PTV for each of the 10 cases in this study are shown in Fig. 2. In the two SCCUP cases, the inferior aspect of the bilateral hippocampal OAR volumes were > 8 mm superior to the superior extent of the PTV, such that the clinical plans already achieved low hippocampal doses (<4.5 Gy mean dose). Therefore, these two cases were excluded from the pair-wise statistical analysis reported in the remainder of this section. Dose reductions achieved by HSRT and BSRT for the 8 NPT cases are summarised in Fig. 3 and Table 2. In one NPT case, overlap between the anterior hippocampal PRVs and the posterior aspect of the primary tumour PTV limited the achievable hippocampal dose reductions. For this case, BSRT (bilateral hippocampi EQD2 D40% = 17.3 Gy) was more effective than HSRT (24.7 Gy) at reducing hippocampal dose from that of the standard clinical plan (33.9 Gy). For the other 7 NPT cases, much greater reductions in hippocampal dose were achievable by both HSRT and BSRT (Table 2). The mean EQD2 D40% for the bilateral hippocampi for all 8 NPT cases was reduced from 23.5 Gy (range 14.5–35.0) for the standard clinical plans, to 8.6 Gy (4.2–24.7) for HSRT (p = 0.001), and to 9.0 Gy (4.3–17.3) for BSRT (p < 0.001).

**Table 2:** Dose statistics for clinical, HSRT, and BSRT treatment plans for all 8 NPT cases.

Dose Statistic	Standard Clinical		HSRT		BSRT		p-value		
	Mean	Range	Mean	Range	Mean	Range	Clinical vs HSRT	Clinical vs BSRT	HSRT vs BSRT
**PTV**									
Conformity Index (CI)	0.77	0.70–0.84	0.78	0.72–0.84	0.75	0.70–0.80	0.613	0.310	0.127
Homogeneity Index (HI)	0.12	0.08–0.23	0.14	0.10–0.24	0.14	0.11–0.24	0.467	0.436	0.975
PTV1 edited D95% (Gy)	61.5	57.0–62.6	61.0	56.7–62.3	61.0	56.9–62.0	0.051	**0.001**	0.839
**Hippocampi**									
**Bilateral hippocampus max (Gy)**	43.6	15.5–62.4	27.1	10.1–45.6	33.7	8.6–59.9	**0.001**	**0.026**	0.169
Bilateral hippocampus mean EQD2 (Gy)	21.5	13.3–32.0	8.4	4.3–23.2	8.7	3.6–17.2	**0.001**	**0.001**	0.939
Bilateral hippocampus D40% EQD2 (Gy)	23.5	14.5–35.0	8.6	4.2–24.7	9.0	4.3–17.3	**0.001**	**<0.001**	0.865
Probability of NCF impairment	0.73	0.42–0.98	0.24	0.10–0.89	0.24	0.08–0.62	**0.001**	**<0.001**	0.944
**Other Brain Structures**									
Whole brain mean (Gy)	12.6	9.3–16.8	10.4	8.2–15.1	9.3	5.6–14.1	**0.006**	**<0.001**	**0.012**
Whole brain max (Gy)	63.6	58.4–69.3	62.8	57.5–70.3	63.6	59.2–69.4	0.179	1.000	0.147
Cerebellum mean (Gy)	34.1	26.3–39.8	29.2	24.7–37.0	28.3	17.8–36.1	**0.033**	**0.037**	0.720
Cerebellum V20Gy (%)	93.6	76.9–98.9	82.5	64.7–94.4	74.9	37.6–97.7	**0.026**	**0.011**	0.284
Cerebellum max (Gy)	58.3	52.4–65.1	55.7	49.7–60.4	57.7	51.9–66.2	**0.041**	0.585	0.282
Brainstem max (Gy)	49.7	48.0–51.6	49.1	46.7–51.8	50.4	49.0–52.1	0.268	0.110	0.070
Brainstem mean (Gy)	36.1	27.3–42.4	32.3	28.5–35.9	29.6	20.9–34.1	**0.011**	**0.001**	0.055
Brainstem V20Gy (%)	91.8	67.5–100.0	89.5	74.3–97.9	75.1	44.0–96.1	0.172	**<0.001**	**0.004**
Left cochlear mean (Gy)	49.4	37.5–65.5	43.4	28.7–66.4	41.7	21.0–65.2	**0.010**	**0.048**	0.394
Right cochlear mean (Gy)	54.1	36.4–65.4	48.3	32.2–65.5	45.2	25.8–66.2	**0.035**	**0.024**	0.201
Left temporal lobe mean (Gy)	24.4	18.6–34.9	20.1	13.7–33.1	20.5	12.9–29.4	**0.012**	**0.019**	0.715
Right temporal lobe mean (Gy)	27.0	19.2–36.6	21.3	14.7–34.0	19.2	9.3–31.4	**0.003**	**0.002**	0.057
Bilateral temporal lobe max (Gy)	63.0	56.3–69.2	62.4	56.6–70.2	62.0	54.8–69.0	0.376	0.342	0.637
Optic chiasm max (Gy)	44.8	34.6–53.4	43.7	32.7–54.0	42.0	30.4–54.0	0.529	0.332	0.563
Left optic nerve max (Gy)	37.4	10.6–52.8	36.7	12.3–54.0	37.8	15.6–54.4	0.782	0.829	0.617
Right optic nerve max (Gy)	37.8	14.0–52.9	38.4	15.0–55.0	35.5	12.7–50.6	0.663	0.270	0.162
**Other OARs**									
Right parotid mean (Gy)	39.3	31.3–48.7	39.4	32.9–50.5	41.6	32.0–53.5	0.926	**0.022**	0.123
Left parotid mean (Gy)	36.3	27.9–45.6	35.4	27.4–45.6	38.2	29.0–51.0	0.097	0.066	**0.012**
Superficial parotids mean (Gy)	29.0	20.8–34.6	29.0	20.4–34.7	32.3	20.9–40.0	0.981	**0.011**	**0.024**
Mandible mean (Gy)	45.6	41.9–51.4	43.6	40.6–47.8	43.6	40.3–48.2	**0.049**	0.065	0.990
Left lens mean (Gy)	4.5	3.0–8.8	4.1	2.9–8.4	5.0	3.0–10.6	0.100	0.133	**0.014**
Right lens mean (Gy)	5.4	2.8–10.8	4.6	2.7–10.6	5.3	2.8–10.5	0.126	0.943	**0.026**
Cochlear PRV max dose (Gy)	59.5	47.1–68.3	57.3	46.7–68.5	56.0	46.8–69.0	0.050	0.074	0.301

Statistically significant results (p < 0.05) are highlighted in bold. Doses are in absolute dose, except those stated for the bilateral hippocampus, that are EQD2 assuming α/β = 2 Gy. The probability of NCF impairment was calculated using the NTCP model of Gondi et al., (2013) [13].

**Fig 4:**
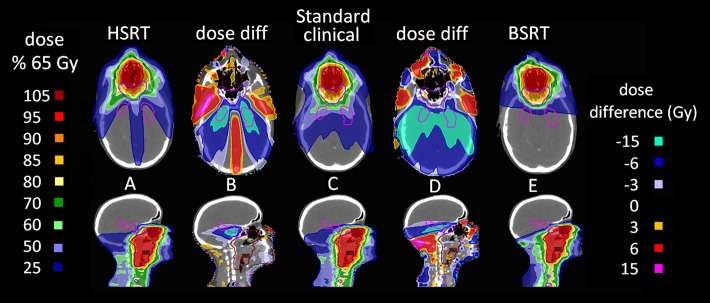
Treatment plans and dose difference maps. (A), (C), and (E) display axial (top) and sagittal (bottom) slices of typical HSRT, standard clinical, and BSRT treatment plans, respectively. (B) and (D) show dose difference maps between (A) and (C), and (E) and (C), respectively.

All HSRT plans were clinically acceptable, both in terms of PTV dose coverage, and OAR sparing (according to clinical objectives listed in S1 Table); selected dose statistics are listed in Table 2. Dose difference maps demonstrate that HSRT resulted in increased dose to some regions of non-contoured normal tissue (Fig. 4B). In order to limit dose to the hippocampi, HSRT typically resulted in increased dose to the maxillary sinuses and the antero-lateral temporal lobes. In 2 of the 8 NPT cases, this increased antero-lateral temporal lobe dose was considered undesirable due to possible risk of neurotoxicity (S3 Fig.).

BSRT was specifically devised to address the increased anterior temporal lobe dose observed with HSRT and to attempt to spare the whole brain. All BSRT plans were clinically acceptable in terms of both PTV coverage, and sparing of OARs (Table 2). Neither HSRT nor BSRT techniques resulted in plans with increased maximum dose to the optic chiasm, optic nerves, brain stem, cerebellum, whole brain, temporal lobes or hippocampi, when compared to standard clinical IMRT (Table 2). Dose difference maps show BSRT results in higher doses to some regions of non-contoured tissue (Fig. 4D). These regions of higher dose correspond to the beam entry paths resulting from the novel beam configuration used for BSRT. However, these dose differences are small (< = 6 Gy), and would not be expected to be of clinical significance. BSRT was successful at reducing the dose to the antero-lateral temporal lobes seen with HSRT (S3 Fig.).

Fig. 1 (E and F) shows a representative example of the achievable hippocampus- and brain-sparing with HSRT and BSRT plans. There were no significant differences between the clinical and HSRT or BSRT treatment plans in terms of CI or HI (Table 2). Cumulative normalised DVHs for various OARs for the 8 NPT cases are shown in Fig. 3. The eye, parotid, mandible, and RVR doses did not differ significantly between the different planning techniques. The mean dose to the cerebellum was significantly reduced from 34.1 Gy to 29.2 Gy for HSRT (p = 0.033), and to 28.3 for BSRT (p = 0.037). Both HSRT and BSRT significantly reduced dose to the whole brain, temporal lobes and brainstem, although the dose reductions were more pronounced for BSRT (Table 2; Fig. 3). HSRT and BSRT reduced the mean whole brain dose from 12.6 Gy to 10.4 Gy (p = 0.006), and 9.3 Gy (p<0.001), respectively.

BSRT significantly reduced the V20Gy for the cerebellum from 93.6% to 74.9% (p = 0.011), and for the brainstem from 91.8% to 75.1% (p<0.001), and these dose reductions are also illustrated by the comparative DVHs (Fig. 3).

The NTCP model of NCF impairment due to brain RT of Gondi et al. [13] predicted that the reductions in the EQD2 D40% of the bilateral hippocampi achieved by HSRT and BSRT for the 8 NPT patients would result in significant reductions in the risk of RT-induced NCF impairment. The NTCP probability was reduced from a mean of 0.78 (min.—max. range 0.48–0.98) to a mean of 0.24 (0.09–0.89) for HSRT (p = 0.001), and to a mean of 0.25 (0.10–0.62) for BSRT (p<0.001) (S2 Fig.).

In terms of deliverability, all 4 of the tested plans (2 HSRT and 2 BSRT) verified successfully on the basis of the delta4 treatment verification procedure (data not shown).

## Discussion

Patients receiving IMRT for HNC often receive biologically significant radiation doses to the brain. The extent of brain irradiation from IMRT for HNC is dependent on the anatomical location of primary tumour [24]. Patients presenting with paranasal sinus tumours typically receive the highest brain radiation doses, but patients with nasopharyngeal tumours also receive relatively high brain doses (Table 1) [24]. For 8 out of the 10 HNC patients studied here, standard clinical IMRT plans delivered sufficient dose to the hippocampi to result in a high probability (∼80%) of subsequent decline in NCF on the basis of an NTCP model.

Patients for this study were selected on the basis that their PTVs encompassed the nasopharynx, therefore resulting in a significant radiation dose bath to the brain using standard IMRT, and these patients are not representative of the general HNC patient population. We deliberately chose these patients so as to present a significant challenge to the brain-sparing IMRT planning process. Conventional IMRT for oropharyngeal primary tumours and associated cervical lymph node metastases may sometimes result in significant hippocampal radiation doses [6], and both HSRT and BSRT are readily applicable to IMRT planning for such patients.

This study demonstrates the feasibility of generating clinically acceptable and deliverable radiotherapy plans for HNC that either specifically spare the bilateral hippocampi (HSRT) or spare the whole brain in addition to the hippocampi (BSRT). Both planning methods result in significant reductions in doses to the hippocampi and, therefore, the probability of subsequent treatment-induced NCF impairment. The freedom to choose beam angles in the non-coplanar fixed-field IMRT plans (BSRT) makes this method particularly effective at sparing the bilateral hippocampi, as well as the remainder of the brain, when treating HNC (Fig. 1). The ability of BSRT to spare most of the brain may be important in reducing disruption to the BBB and, thereby, reducing access to the brain for concomitantly delivered chemotherapy agents. The relatively complex non-coplanar beam arrangement used by BSRT inevitably results in longer treatment delivery times than standard IMRT or HSRT, but the potential reduction in late toxicity may justify this cost for selected HNC patients. Whether the degrees of hippocampal and brain radiation dose-sparing achieved by HSRT and BSRT are sufficient to result in reduced late neurotoxicity and preservation of NCF requires testing in prospective clinical studies. The ability of HSRT and BSRT to preserve NCF may depend in part on the relative contributions of radiotherapy and chemotherapy to late neurotoxicity. However, whilst the dosimetric considerations presented here give rise to concern regarding NCF outcomes for patients treated using standard (chemo)-IMRT for HNC, there is at present a paucity of clinical data on NCF outcomes for such patients [24]. Additional data on NCF outcomes for HNC patients receiving modern standard treatment are needed, as a prelude to interventional studies testing NCF-sparing interventions [24].

For 7 of the 8 NPT cases re-planned in this study, a significant reduction in hippocampal dose was achievable using both HSRT and BSRT. Both methods resulted in a lateral bowing of dose at the superior extent of the PTV and this was typically more pronounced using HSRT. For the remaining NPT case there was overlap between the hippocampal PRV and the PTV (case 1; Fig. 2). In this case the bilateral hippocampus EQD2 D40% was reduced from 33.9 Gy to 24.7 Gy (reducing the probability of NCF impairment from 0.98 to 0.89) for HSRT, and to 17.2 Gy (reducing the probability of NCF impairment to 0.62) for BSRT. Therefore, in situations where there is close proximity between the hippocampi and the PTV, BSRT would appear to be the preferred planning intervention.

For the two cases of SCCUP, the doses to the bilateral hippocampi for the standard clinical plans were already sufficiently low to result in low probabilities of post-treatment NCF impairment (NTCP of 0.05 and 0.11 for cases 9 and 10, respectively). The bilateral hippocampus dose was correlated with the axial distance between the inferior extent of the hippocampus OAR volume and the superior extent of the PTV. In both the SCCUP cases, dose to the hippocampi mainly resulted from scatter, meaning that it was not possible for the co-planar HSRT planning method to meaningfully reduce hippocampal dose without compromising PTV coverage. However, in both of these cases, BSRT did succeed in reducing hippocampal doses, as well as doses to the whole brain, brainstem and cerebellum.

Analysis of dosimetric data from the PARSPORT trial has shown that the excess fatigue in the IMRT arm is associated with increased dose to the posterior fossa, and specifically to the cerebellum [6]. The results presented here show that it is possible to spare the posterior fossa, including the cerebellum, particularly by using BSRT. A clinical trial of BSRT for HNC would allow us to test the hypothesis that such dose reductions are sufficient to result in reduced acute fatigue. Additionally, such a study would provide data on the impact of BSRT on NCF outcomes.

This study focussed on generating brain-sparing RT plans for photon-based HNC radiotherapy. The potential benefits of proton beam therapy (PBT) for HNC are currently being investigated [29]. Due to the rapid dose fall-off beyond the Bragg peak, intensity-modulated PBT (IMPT) has potential for generating highly brain-sparing treatment plans for HNC, and our results indicate that a minority of HNC patients may specifically benefit from IMPT in situations where the PTV and hippocampal PRVs overlap, or are in close proximity.

Finally, our results highlight the need for the collection of additional prospective data on NCF outcomes for HNC patient populations treated with radical (chemo)-RT. Such studies will provide the rationale for subsequent clinical studies of manoeuvres, such as those described here, to reduce the impact of IMRT for HNC on NCF.

## Supporting Information

S1 Fig Illustration of the non-coplanar beam arrangement for a typical BSRT plan, including anterior superior oblique, and posterior inferior oblique beams. Combinations of gantry angles and couch twists were chosen such that all beams were deliverable, without collision, on Elekta linacs.
(TIF)

S2 Fig Bilateral hippocampal EQD2 D40% data for standard clinical plans (red), HSRT (green), and BSRT (blue) on the NTCP model of NCF after brain RT (as measured by the Wechsler Memory Scale-III Word Lists delayed recall at 18 months post-RT) of Gondi et al., (2013) [13] for the 8 NPT cases. (TIF)

S3 Fig Illustration of the effect of HSRT on dose to the antero-lateral temporal lobes (left panel, arrows), relative to the standard clinical plan (centre panel). BSRT succeeds in eliminating this additional temporal lobe dose as well as further reducing the whole brain dose (right panel, arrows).
(TIF)

S1 Table Clinical dose statistics and objectives for targets, organs at risk (OARs), and planning-at-risk volumes (PRVs). (PDF)
