# Supplementary figures and images for: Brain-Sparing Methods for IMRT of Head and Neck Cancer
Source: PLoS One. 2015 Mar 17;10(3):e0120141. doi: 10.1371/journal.pone.0120141 (PMC4364536; doi:10.1371/journal.pone.0120141)

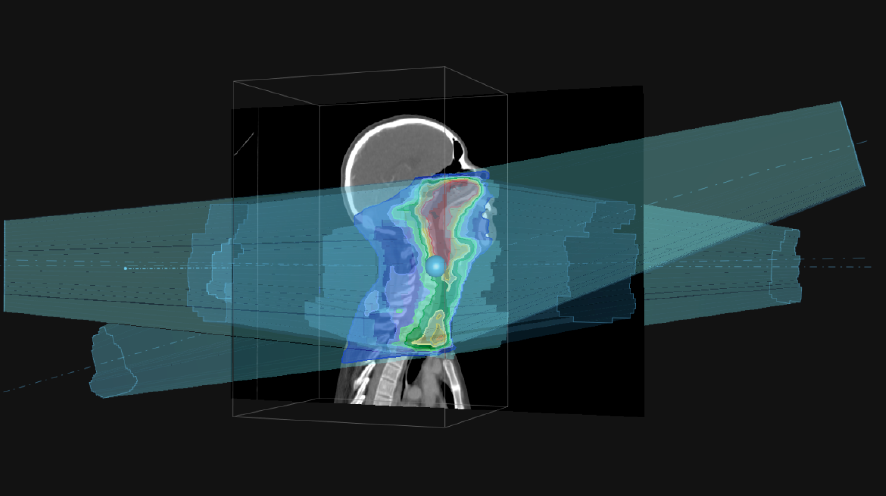

Supplement: S1 Fig — Combinations of gantry angles and couch twists were chosen such that all beams were deliverable, without collision, on Elekta linacs. (TIF) [file pone.0120141.s001.tif]

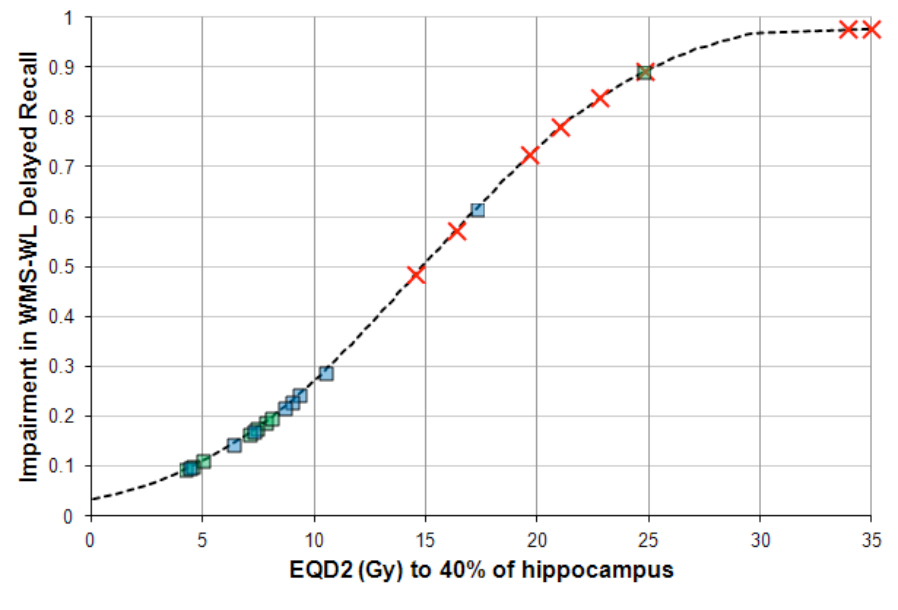

Supplement: S2 Fig — (TIF) [file pone.0120141.s002.tif]

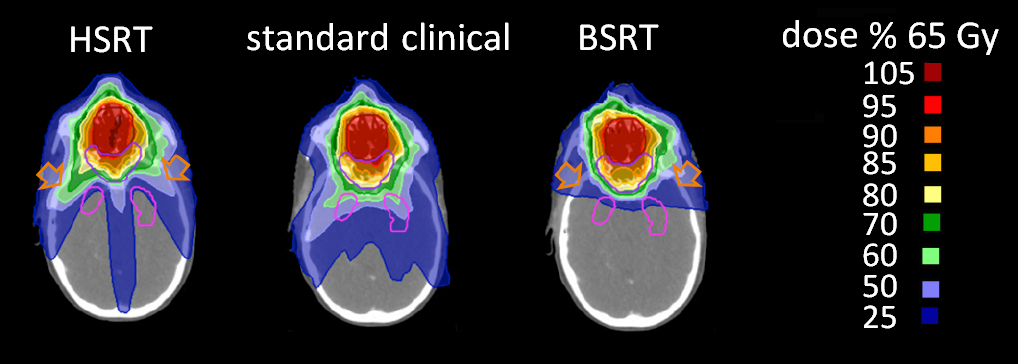

Supplement: S3 Fig — BSRT succeeds in eliminating this additional temporal lobe dose as well as further reducing the whole brain dose (right panel, arrows). (TIF) [file pone.0120141.s003.tif]
