# Supplementary material for: Brain-Sparing Methods for IMRT of Head and Neck Cancer
Source: PLoS One. 2015 Mar 17;10(3):e0120141. doi: 10.1371/journal.pone.0120141 (PMC4364536; doi:10.1371/journal.pone.0120141)
Supplement: S1 Table — (PDF) [file pone.0120141.s004.pdf]

Structure	Dose statistic	Optimal	Required
PTV1 edited	Dose to 99%		> 58.5 Gy
PTV1 edited	Dose to 95%		> 61.8 Gy
PTV1 edited	Dose to 50%		65 +/- 1 Gy
PTV1 edited	Dose to 2 %		< 69.5 Gy
PTV2 edited	Dose to 99%		> 48.6 Gy
PTV2 edited	Dose to 95%		> 51.3 Gy
PTV2 edited	Dose to 50%		54 +/- 1 Gy
Bilateral parotids	Mean dose	< 24 Gy	< 30 Gy
Superficial parotids	Mean dose	< 24 Gy	< 30 Gy
Spinal cord	Maximum point dose		< 46 Gy
Spinal cord PRV	Maximum point dose		< 48 Gy
Brain stem	Maximum point dose		< 50 Gy
Brain stem PRV	Maximum point dose		< 55 Gy
Optic chiasm	Maximum point dose		< 54 Gy
Bilateral optic nerves	Maximum point dose		< 55 Gy
Bilateral lens	Mean dose		< 6 Gy
